# Single Incision Laparoscopic Surgery (SILS) in Small Animals: A Systematic Review and Meta-Analysis of Current Veterinary Literature

**DOI:** 10.3390/vetsci8080144

**Published:** 2021-07-28

**Authors:** Luca Lacitignola, Marta Guadalupi, Federico Massari

**Affiliations:** 1 Dipartimento dell’Emergenze e Trapianti di Organi (D.E.T.O.), Sezione di Cliniche Veterinarie e Produzioni Animali, Università Degli Studi di Bari “Aldo Moro”, 70010 Bari, Italy; marta.guada92@gmail.com; 2 Clinica Veterinaria Nervianese, 20014 Nerviano, Italy; federicomassari@me.com

**Keywords:** laparoscopy, single incision laparoscopy, dog, cat, small animals

## Abstract

In veterinary surgery, single incision laparoscopic surgery (SILS) techniques have been described since 2009, and, in recent decades, many authors have reported the application of SILS in small animals, thus, promoting the wide dissemination of this novel approach among veterinary laparoscopists. The aim of this literature review is to provide a critical evaluation of the scientific reports on SILS in the field of small animal laparoscopic surgery. A comprehensive literature review was performed including from 1 January 2009 to 1 July 2020. The following data were recorded from each study: the design, year of publication, surgical procedure, species, number of animals included, and surgical time. The type of SILS technique and type of control group technique were evaluated. In total, 90 articles were identified through database searches and manual searches. The qualitative analysis showed that most of the articles were retrospective studies, without a control group or case series. A meta-analysis was performed on the eight controlled studies, showing that SILS ovariectomy and gastrointestinal procedures had a comparable surgical time to multiport techniques. The study of the articles available in the veterinary literature did not allow for an adequate meta-analysis of the published results, especially regarding post-operative pain, evaluations of surgical times, and post-operative complications in comparison to multiport techniques. Therefore, veterinary surgeons who want to employ these techniques must consider the real advantages of SILS techniques.

## 1. Introduction

Single Incision Laparoscopic Surgery (SILS) is a minimally invasive endosurgical technique that is increasingly being performed by surgeons in human medicine all over the world. The SILS technique consists in reducing the number of portal sites resulting in less surgical trauma and pain and improving cosmetic outcomes [1]. However, SILS is associated with a number of challenges, such as a loss of triangulation and, therefore, conflict between instruments. Due to these technical difficulties, the technique can result in a longer operative time and longer learning curve, compared to multiport techniques [2].

In veterinary surgery, the SILS technique has been described since 2009 [3], and in recent decades, many other authors have reported the application of SILS in small animals, thus, promoting the wide-dissemination of this novel approach among veterinary laparoscopists. Many SILS platforms are currently available among the surgical armamentaria. Recently, they have been widely reviewed in the human surgery field [1] and veterinary applications [4,5].

Authors have described the available platforms and instrumentations, as well as their application. In particular, the use of the SILS port ™ (Covidien, Mansfield, MA, USA), EndoCone port (Karl Storz, Goleta, CA, USA), the GelPOINT access system (Applied Medical, Rancho Santa Margarita, CA, USA), and Wound Retractor with Latex Glove and Finger Ports have been reported.

The aims of this literature review are to provide a critical view of the scientific reports on SILS in the field of small animal laparoscopic surgery and to perform a meta-analysis on studies investigating SILS versus other laparoscopic techniques to verify the efficacy in decreased surgical time and pain level in small animals. 

## 2. Materials and Methods

### 2.1. Search Strategy, Selection of Studies, and Data Evaluated

The studies were selected according to the international principles of preferred reporting items for systematic reviews and meta-analyses (PRISMA). [6]

A comprehensive literature review was performed on 1 July 2020, by using the Web of Science databases. The databases queried for searching included Web Of Science and Medline. Search terms included the following keywords: “single-incision laparoscopic surgery dog OR single-incision laparoscopic surgery cat OR single port laparoscopy dog OR single port laparoscopy cat OR single port laparoscopic assisted surgery dog OR single port laparoscopic assisted surgery cat”. The search range was from 1 January 2009 through 1 July 2020. Further additional references were hand searched in the reference section of each paper.

The inclusion criteria included English-language articles that provided a description of SILS or laparoscopic-assisted surgery in dogs and cats. The study types included Randomized Controlled Studies (RCT), Retrospective Studies (RS), Case Series Studies (CS) and Case Reports (CR), Clinical Trials (non-randomized, non-controlled) (CT), and Controlled Clinical Trials (CCT). The exclusion criteria included narrative review articles, editorials, and studies involving other species. Studies that involved both dogs and cats but separated information by species, were not excluded. After the research strategy was applied, the author assessed the articles by title and abstract for inclusion as an initial screening. The full text of the identified articles was assessed, thus, producing the final selection of articles included for review.

The following data were recorded from each study: design, year of publication, surgical procedure (ovariectomy, ovariohysterectomy, cryptorchidectomy, gastropexy, gastrointestinal explorative surgery, splenectomy, and combinations of these), species, number of animals included, and surgical time. Moreover, the type of SILS technique and type of control group technique were evaluated. 

The level of evidence was classified according to previously published scales. Refs. [7,8] Table 1.

### 2.2. Statistical Methods

The meta-analysis was performed by MedCalc Statistical Software version 16.4.3 (MedCalc Software bvba, Ostend, Belgium). For meta-analysis of the studies in which the comparison of means between SILS data versus other techniques described, the software employed the Hedges g statistic as a formulation for the standardized mean difference under the fixed effects model. Next, the heterogeneity statistic was incorporated to calculate the summary standardized mean difference under the random effects model. The significance level was fixed at *p* < 0.05.

## 3. Results

### 3.1. Study Characteristics

**Figure 1:**
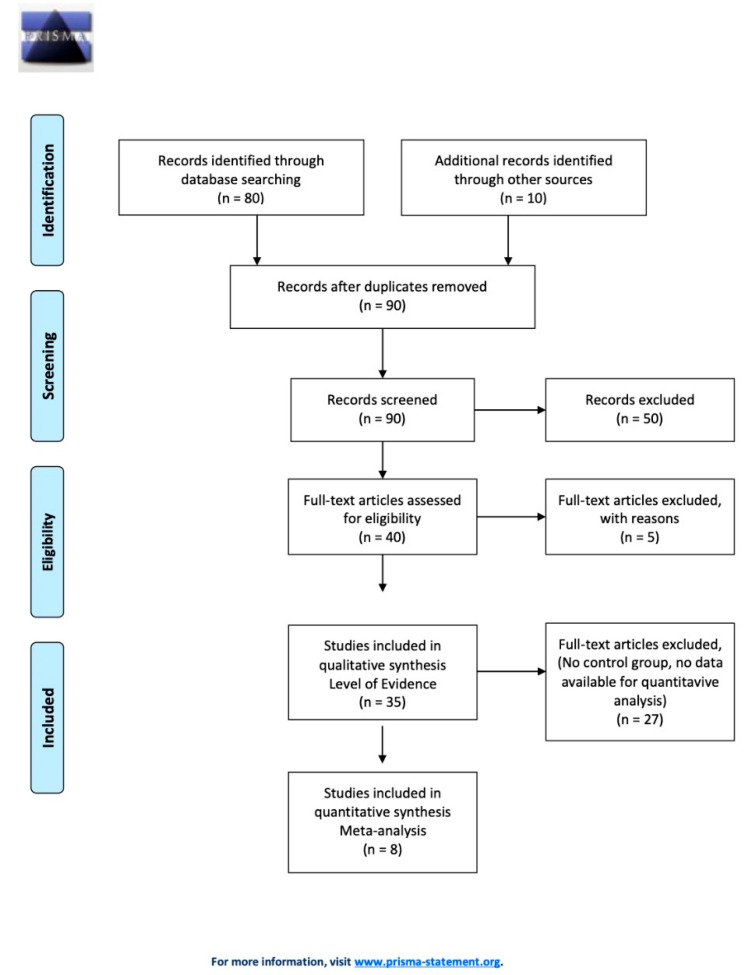
Study selection flow chart.

In total, 80 articles were identified through database searches, and ten were added from the manual searches. No duplicates were founded, and the manuscripts were screened, leaving 40 studies that met the inclusion criteria for this review. No systematic reviews articles were found. The most common reasons for exclusion were the article review design, other species than dog and cat, and human studies. Additional reason for exclusion included non–English-language articles. Five studies were excluded after the full text was assessed, and 35 studies were eligible for data extraction. Figure 1 shows the selection process.

**Figure 2:**
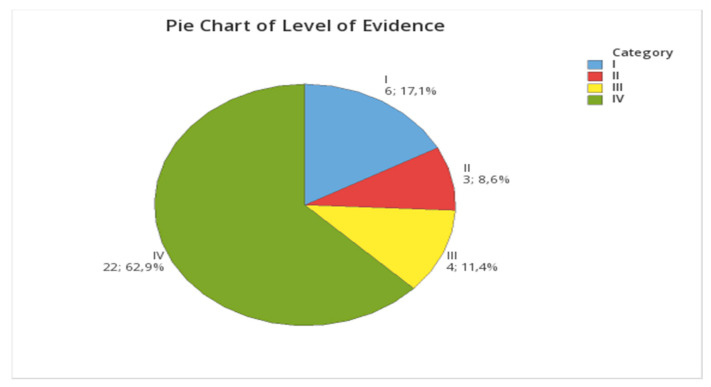
Pie chart of the level of evidence. I, Randomized controlled trial; II, Prospective cohort study patients compared with a control group of patients treated at the same time and institution; III, Case control study and retrospective cohort study; and IV, Case series (no control group or historical control group).

A description of the study method or design was incomplete or not mentioned in 9/35 articles and had to be assigned by the author. Among the 35 studies selected, there were eight (22.9%) Case Series CS, five (14.3%) Randomized Clinical Trials (RCT), 17 (48.6%) Retrospective Studies (RS), one (2.9%) Clinical Trial (non-randomized, non-controlled) CT, two (5.7%) Controlled Clinical Trials (CCT), and two (5.7%) Case Reports (CR), as shown in Figure 2. Fourteen studies (40%) included the control population, as shown in Table 2.

In total, 1001 dogs and 137 cats were described within 35 studies, with a median of 18 (range, 1–278) dogs and 17.5 (range, 1–42) cats per study. Fourteen (40%) studies included the control population, as shown in Table 2. 

### 3.2. Techniques

#### 3.2.1. Operative Telescope

The operative telescope (OT) used was a 10-mm-diameter operating laparoscope, with a working channel (HOPKINS Optik 0°, 10 mm, Model 26038 AA, Karl Storz GMBH & Co. KG, Tuttlingen, Germany). The operative channel allows for the insertion of 5-mm instruments. The technique uses an 11- or 12-mm trocar inserted through a skin incision performed at a site 1–2 cm below the umbilicus for the telescope insertion. This technique has been described for OVE [3,9,10,11,34], OVH [23,35] and for GPX in combination with OVE [28]. In all studies, the ovary suspension was performed by an external suspension suture. In OVH surgery, the entry site was established in the median prepubic region, and the uterine ligation was performed outside the abdominal cavity by bipolar coagulation [23].

#### 3.2.2. SILS Port System ™

The SILS Port (Medtronic, Fridley, MN, USA) is a foam port composed of a compressible elastic polymer designed to accommodate three cannulas and has a separate insufflation tubing. The flexible port construction facilities easy placement of the incision to maintain pneumoperitoneum and allows for maximal maneuverability of the instruments. The port can be used with three 5-mm cannulas or two 5-mm cannulas and one 10-mm or 12-mm cannula to accommodate a variety of laparoscopic instruments. 

The SILS port is inserted through a 2- to 3-cm long mini-laparotomy. Despite its original single use purpose, the SILS port can be re-sterilized using appropriate methods [17]. The surgical procedures in which this port system has been employed are OVE [11,16,20], ovaryhysterectomy (OVH) [17], gastropexy (GPX) [21], OVE+GPX [14,21], gastrointestinal laparoscopic assisted surgeries [15,22], Cryptorchidectomy [19], and ovarian remnant syndrome (ORS) [18].

#### 3.2.3. Endocone

The EndoCone (Karl Storz GMBH & Co. KG, Tuttlingen, Germany) is a stainless steel reusable conical port with a removable bulkhead and flanged leading edge. The port is inserted in through a 3-cm mini-laparotomy, which is created in advance for port insertion.

One advantage of the EndoCone is that the superior cap can be opened repeatedly during the procedure, allowing for re-insufflation of the abdomen at any time during the procedure. Another major benefit of the EndoCone is its ability to be sterilized in an autoclave.

#### 3.2.4. Ring Wound Retractor (WR)-Based Port System

Recently, many SILS platforms have been based on a combination of a ring wound retractor and multiport fabric systems. The retractor can be equipped with a cap for laparoscopic instruments or it can be “customized” using Single or Multiport fabric systems available in the market, such as Gel Ports, Triports, “glove port”, etc. The wound retractor port system is placed through an approximately 25-mm to 70-mm-long skin incision, accordingly to the retractor size. After the laparotomy, the inner ring is inserted into the abdominal cavity and advanced using the forceps. Thereafter, the outer ring is adjusted to install the inner ring inside the abdominal wall. 

The outer ring is rolled up until the right tension is achieved, fixing the inner ring and creating retraction on the laparotomy. The cap is applied to the outer ring, and the valve is connected to inject CO^2^ gas and, thus, obtain the desired intra-abdominal pressure. Deriving from the human surgery application, the glove port system was introduced into veterinary applications to reduce the costs associated with equipping specific fabric platforms. 

Additionally, the combination of a WR and SILS port system was reported to offer advantages in terms of the radial retractor and SILS port. The reported advantages of this technique are that the surgeon can interrupt the pneumoperitoneum at any time by removing the cap, performing extracorporeal procedures, or removing a large specimen from the abdomen more easily, and then re-inserting the cap, re-insufflate the abdomen, and continue the procedure in the laparoscopic environment.

The wound retractor is also a low-cost alternative to other port devices and avoids the further costs associated with the use of disposable retrieval bags or morcellators. Furthermore, although it was designed as a disposable device, the wound retractor with the cap can be reused several times after sterilization, as previously described [38].

### 3.3. Surgical Procedures

#### 3.3.1. Ovariectomy (OVE)

OVE was the most frequently surgical procedure performed in the articles investigated. 

Duprè et al. [3] tested the feasibility, surgical time, and complications associated with SILS OVE surgery in dogs. The authors compared the single portal with the OT versus two portals. No significant difference was found in the perioperative complication rates and the mean total surgical time between the SILS procedures (21.07 min) and multiport procedures (19.06 min). Notably, a limitation of the operating laparoscope was the reduced degrees of freedom to work and limited movements. Moreover, the authors stated that a body weight of <7 kg may represent a potential limitation. 

Another study was performed by Case et al. [9]. This study described the OVE in dogs, performing SILS by OT versus the three cannulas multiport technique and two cannulas multiport approach. The results of this study were that the surgical time was significantly longer using OT SILS (29.7 ± 5.6 min) than that using two cannulas (18.2 ± 4.4 min) or three cannulas (19.3 ± 3.4 min). The complication rates were not significantly different among the groups. 

The authors also found that the pain score was significantly lower using two cannulas compared with using three cannulas but the pain score in the one-cannula group did not differ significantly from the other groups. The main limitations of SILS OT were the use of smaller instruments and a lack of independent maneuverability of the instruments and the laparoscope. Therefore, the authors concluded that the two-cannula technique allowed for complete independence of the endoscope from the instrument while maintaining the triangulation capability. The one-cannula technique limits the independent maneuverability of the instrument only in-depth, increasing the surgical time and making the procedure technically more difficult.

In the study performed by Kim et al. [10], the use of OT was studied in cats, evaluating the surgical time, complications, and pain. The results of the study showed a surgical time of 23 min and seven seconds, and no intra- and post-operative complications were encountered. No cats needed rescue analgesia within 24-h post-surgery. Among the advantages of the technique, the authors reported that SILS OVE with OT in cats could provide an improved visualization of the ovary and associated mesovarium, and reduced manipulation.

Runge et al. [13], proposed a single port access. The authors described this laparoscopic entry technique for canine OVE, reporting the complications and outcomes. A 1.5–2-cm ventral median skin incision was made midway between the caudal aspect of the xiphoid and the umbilicus. Using the Hasson abdominal access technique, a 5-mm blunt laparoscopic trocar was inserted into the abdomen, and a pneumoperitoneum was created. 

Using minimal blunt dissection, a tunnel was created 2-cm laterally on either side of the initial central trocar using a Kelly hemostat. Through those tunneled paths, two 5-mm trocars were inserted into the tunnels caudolaterally. The procedures were performed in a mean time of 52.5 min. The authors concluded that the laparoscopic entry technique could be used in dogs, although complications can occur if the trocar placement is too consolidated within the initial skin incision.

Binder et al. [34] reported the post-operative complication rate, pain score, and owners’ satisfaction rates of SILS OVE in dogs using an OT. The results of this study were that SILS OVE with OT had minor complication rates compared to other laparoscopic techniques and had the advantages of a low post-operative pain level evaluated by the owner. However, a high rate of wound complications (12.9%) was found. Notably, main post-operative complication (5.3%) was that the dogs removed their sutures. 

The overall percentage of patients with pain signs was only 7.7% for more than 24 h after surgery. However, the authors underscored that the major advantage of laparoscopic surgery was a low incidence of post-operative pain probably due to the reduced incision length and, thus, the reduced level of tissue trauma. The main limitation assured in this study was that the pain assessment was evaluated by the owner and, therefore, did not rely on objective criteria.

OVE was also largely investigated by employing this procedure with the SILS port platform. Coisman et al. [17] evaluated the feasibility of SILS OVE in cats using the SILS port system. The aim of this study was to compare the surgical time, complications, and post-operative pain after SILS OVE using a bipolar vessel sealing device versus extracorporeal suture, and open OVE. The results of the study were a median surgical time of 25.5 min for the SILS and vessel sealing device group; 71 min for the SILS OVE and extracorporeal suture group; and 17.5 min for the open-OVE group. The authors concluded that the SILS port system was a feasible method for OVE in cats. SILS OVE using an extracorporeal suture is more time-consuming and is associated with more complications than either the bipolar vessel sealing device or open-OVE methods. 

Manassero et al. [12] investigated the role of transabdominal suspension suture during SILS OVE with the SILS port system in dogs. The authors evaluated the surgical time and complications rate. The results of this study were that the mean surgical time was similar between the groups, with a minor rate of splenic injury in the installation phase. 

The authors concluded that OVE with the SILS Port device, since it is performed with two instruments, might be performed without a transabdominal suspension suture. However, the main challenges reported were limited triangulation and instrument interference and, consequently, reduced maneuverability. An advantage encountered in this study was the easy retrieval of the removed ovary through the 3-cm-long incision. Moreover, if the ovaries were lost during the extraction, they could be easily retrieved. 

Gonzalez-Gasch and Monnet [21], investigated the duration of surgery, the frequency of complications, and the frequency of open conversion for elective surgeries performed with SILS and a multiple port access system (two cannulas). The results of the study were the significantly shorter time for SILS OVE (43 min) versus the multiport setup (70 min). The intraoperative complications rate was significantly lower for SILS compared with the multiport approach. Moreover, the main complication observed was incisional infection. The authors ascribed to obviation of the use of a Veress needle, the decreased surgery time with SILS. 

Tapia-Araya et al. [24] performed a controlled trial, studying the feasibility and safety of SILS and three-portal laparoscopic OVE in dogs. The results of the study were that the mean total surgical duration was slightly longer using SILS (36.6 ± 3.5 min) than multiport OVE (32.0 ± 3.0 min); however, the differences were not significant. The authors reported that SILS was more technically demanding because of the loss of triangulation, reduced maneuverability, and instrument collisions. Moreover, the authors noted the reduction of traction capability, potentially resulting in lacking to manage intraoperative bleeding. However, the authors reported a faster recovery in all cases and no post-operative complications. The study did not evaluate the pain level between the two techniques.

Corriveau et al. [33] compared the outcomes for laparoscopic OVE and laparoscopic-assisted OVH in dogs. All OVH cases, except one, were performed with a multiport laparoscopic technique, whereas 99 of 147 (67.3%); OVE cases were performed with a SILS technique. Among single-port cases, SILS port was used in 91 of 99 (91.9%) cases. The surgery time for the SILS vs. multiport techniques was 50 and 67 min, respectively. The results suggested that the short- and long-term follow-up were similar for SILS OVE or multitrocar OVH; however, SILS OVE showed a shorter surgery time, and the owner’s satisfaction was detected in both groups.

#### 3.3.2. Ovariohysterectomy (OVH)

In 2015, Silva et al. [23] compared the surgical time, complications, and technical difficulties associated with transvaginal total natural orifice transluminal endoscopic surgery (NOTES) and single-port laparoscopic-assisted and conventional OVH in bitches. In the NOTES and SILS procedures, the OT technique was employed. The authors observed a mean surgical time of 25.7 ± 6.8 min for NOTES and 23.1 ± 4.0 min for the SILS groups, and 34.0 ± 6.4 min in the OPEN group. 

The surgical procedure was divided into seven stages; the longest phase was the approach to the uterine body in the NOTES group and abdominal and cutaneous sutures in the OPEN group. The authors noted that the length of the cannula was insufficient to perforate the vagina serosa and enter the abdominal cavity in 50% of the cases. All patients in the NOTES group presented mild non-painful vulvar swelling. Seroma formation was observed postoperatively with laparoscopic-assisted OVH. The authors acknowledged, as an advantage, the fact that, in the NOTES group, the incision is located within the vaginal canal, which avoids complications, such as self-trauma, evisceration, and the need for protective collars.

Coutinho et al. [35] studied the surgery time, complications, pain, and post-operative inflammatory response caused by SILS OVH and traditional mini-celiotomy with a snook hook. The SILS OVH was performed by OT. A cervix suture was performed on the outside the body. The authors observed a surgical time of 41.1 ± 5.9 min, and a complication rate of 17% in both groups. Rescue analgesia in the Post-operative phase and the C-reactive protein concentration were similar between groups. However, the animals in the SILS OVH group were fed earlier and presented lower stress levels. 

Recently, Bydzovsky et al. [38] described an SILS laparoscopic-assisted OVH surgery with a modified glove-port technique in dogs. The retractor was positioned at 2/3 of the distance between the umbilicus and the pubis, which enabled all the surgical steps to be completed through the same hole. The median total duration of surgery was 24 min (range, 17.5–39.5; mean, 25.73; SD, 6.12). The intraoperative complications were minor; however, wound complications occurred in 29% of dogs. Another reported challenge in this study was the intra-abdominal interference of instruments, although this never impeded surgery. The authors attributed significant maneuverability to the flexibility of the glove port. The open approach used for the glove-port technique dramatically limited the risks of iatrogenic-entry-related complications. 

#### 3.3.3. Pyometra

The pyometra in the bitch was initially treated with a laparoscopic-assisted approach using the three-multiport technique, inserting a WR in the caudal port, and performing a cervix closure extracorporeally. [16]. More recently, Wallace et al. [25] used a SILS port system to perform an ovary dissection and continue the cervix closure extracorporeally, removing the SILS port and elective OVH surgery. The results of the study showed a median surgical time of 85 min (range 40–110). 

The median uterine body diameter was 2.2 cm (range 2–3.9). As a minor intraoperative complication, the creation of an additional port in one dog to exteriorize the uterine body was reported, because the first portal was created too cranially. Conversion to an open procedure was described in one dog because of uterine rupture. The authors concluded that the appropriate port placement was essential for the adequate visualization and dissection of the ovarian pedicle and to avoid having in place an additional caudal port. The authors considered this technique feasible in dogs with a uterine body diameter greater of up to 4 cm. 

Conversely, in 2016, Becher-Deichsel et al. [27] described the use of WR with a glove port, instead of a SILS-port. Using a median incision length of 5.0 cm, the authors performed the procedures in a median surgical time of 57 min. In two dogs, the incision was lengthened to exteriorize the uterus. The main post-operative complication that occurred was the related wound healing. The authors highlighted the easy exteriorization of uterus through the WR. Additionally, the absence of a cannula protruding into the abdomen decrease the risk of damage of abdominal organs. The authors concluded that the technique was feasible treatment of canine pyometra with a diameter of up to 7 cm.

#### 3.3.4. Cryptorchidectomy

Runge et al. [19] described SILS cryptorchidectomy with the use of several different platforms: the SILS port system, Triport System, and EndoCone Port in dogs and cats. The results of the study were a median surgical time of 39 min (range 15–70 min) and 1.5- to 3-cm abdominal incision length. The SILS technique was associated with a low complication rate and provided a potentially less invasive alternative to traditional open and multiport laparoscopic techniques. The reported advantages of SILS observed in this study was that each of the single-port devices enabled specimen retrieval at any point during the surgical procedure, and the pneumoperitoneum could be re-established quickly and efficiently. No comparisons among ports were made in this study.

#### 3.3.5. Ovarian Remnant Syndrome (ORS)

Ovarian remnant syndrome was removed laparoscopically, by means of either a three-port or SILS technique with the patient in dorsal recumbency. Six procedures were performed with a standard three-port technique, and one was performed with a single-port technique. No data were available about the advantages or disadvantage of the different procedures. [18]

Percival et al. [42] described the removal of ovarian remnants in 13 dogs with the use of the SILS-port platform. The authors reported a surgical time with a median of 45 min (range, 30–90 min). In one dog, a supplementary port was placed cranially because the SILS-port was initially located too caudally and did not allow for proper dissection and tissue manipulation. No other complications were mentioned.

#### 3.3.6. Gastropexy (GPX)

Gastropexy surgery was also largely performed with SILS. Gonzalez-Gasch and Monnet [21] investigated the duration of surgery, the frequency of complications, and the frequency of open conversion in dogs undergoing laparoscopic-assisted gastropexy using a SILS-port system versus laparoscopic-assisted multiport technique. The surgery duration was significantly shorter in the SILS-port group (43 min) compared with that of the multiport procedures (61 min).

In 2016, Stiles et al. [29] described the technique, clinical findings, and short-term outcomes in 14 dogs undergoing laparoscopic-assisted incisional gastropexy with Endocone. The authors reported a mean surgical time of 76 min. Minor complications were gas leakage in 2 of 14 dogs and a surgical site infection in a dog. The results suggested that SILS GPX with Endocone was feasible and effective. The authors verified that an appropriate incision length can prevent a difficult placement (if the incision is too small) or gas leakage (if excessively long (>30 mm)).

#### 3.3.7. Combining Gastropexy with Ovariectomy

Many authors associated OVE surgery with prophylactic gastropexy. Runge et al. [14] described a technique for SILS GPX + OVE using the SILS port system to evaluate the short-term outcomes in dogs. The laparoscopic-assisted incisional gastropexy was performed after ovariectomy at the multi-trocar port insertion. The procedures were completed in a median surgical time of 65 min. 

The intra-operative complications included an incorrect multi-trocar port placement location and splenic laceration requiring conversion to open laparotomy. Two dogs had incisional seroma formation on 14 days follow-up. The authors concluded that the SILS GPX + OVE was a feasible procedure. The authors recommended careful and accurate initial SILS port placement as necessary for improving operative viewing and prevent splenic injury. Moreover, SILS technique requires a distinct learning curve.

In another study, an OT telescope was described for procedures combining OVE and GPX [28]. Percutaneous laparoscopic gastropexy was performed in dogs using resorbable, barbed suture material in combination with a single-port access ovariectomy. The surgery time and intraoperative, post-operative, and follow up complications were reported. The median surgery time was 73 min, and no complications were observed. The authors described difficulties in the simultaneous handling of the instruments and laparoscope and the identification of the gastropexy site on the stomach wall.

#### 3.3.8. Splenectomy

Wright et al. [30] described the operative technique and perioperative outcomes for laparoscopic-assisted splenectomy in dogs using the SILS port and GelPOINT. The procedures were performed intra- or extracorporeally in 14/18 patients, including abdominal organ biopsies, esophagostomy tube placement, laparoscopic-assisted gastropexy, percutaneous cystolithotomy, ovario-hysterectomy, typhlectomy, omental mass resection, right hemithyroidectomy, and bone marrow biopsy. The procedures were completed in a mean of 60 min (range, 45 to 130 min). The SILS platform was placed through a 2.5-cm laparotomy placed caudally to the umbilicus.

The technique consisted of abdominal exploration and laparoscopic staging, followed by abdomen desufflation and removal of the SILS platform used. The sub-umbilical incision was extended to a mini-laparotomy, depending on the size of the spleen. In two dogs in which the laparoscopic access device had been used, the extension of the incision was not required, because a 5- to 7-cm-long mini-laparotomy incision had already been used. In this study, the device was found to be easy to place and facilitated abdominal access and atraumatic exteriorization of the spleen through the mini-laparotomy incision, and potential contamination with neoplastic tissue during specimen retrieval.

In 2018, Mayhew et al. [36] described the technique and reported the complications and outcomes for single-port laparoscopic splenectomy in dogs. The SILS port and GelPOINT platform were used in this study. The authors recommended this technique in cases of smaller dogs with modestly-sized splenic masses or diffuse splenic disease. The authors recommended the removal of the SILS port and placement of a wound retraction device after splenic separation from the mesentery was completed. The use of a retrieval bag was recommended to facilitate splenic retrieval. In the case of the GelPOINT access system, there is no requirement to place a separate wound retractor if this single- port device is used.

#### 3.3.9. Laparoscopic Assisted Gastrointestinal Surgery

In 2013, Case J.B and Ellison G. [15] investigated the surgical time and short term outcomes in dogs and cats undergoing SILS laparoscopic-assisted intestinal surgery using the SILS port system or EndoCone port. The procedures described were incisional biopsy, enterotomy, and foreign body removal, resection and anastomosis for foreign bodies or intussusception, and omentalization.

The authors completed the procedures in a mean of 120 min. SILS laparoscopic–assisted procedures were reported by the authors in dogs and cats, which were found to be feasible and effective in selected cases. As the complete abdominal exploration was limited using the SILS approach, the authors considered preoperative imaging to be of great importance. A technical complication encountered using the single-incision approach, especially with straight instruments, was the collision of the instruments.

Rubin et al. [22] described a case of a dog laparoscopic-assisted, mid-jejunal resection and anastomosis using a single-incision laparoscopic surgery port (SILS port system). In this single reported case, a short post-operative hospitalization time and no major anesthetic or intraoperative complications were reported. The use of the SILS port, in this case, allowed for the minimization of the surgical wound, while allowing for intestinal resection and anastomosis.

Baron et al. [31] showed the surgical technique and evaluated the short-term outcome of laparoscopic small intestinal exploration and organ biopsy with a wound retractor device (WR) in cats. Full-thickness, small-intestinal biopsies were obtained extracorporeally via the WR. In 59.6% of cases, a combination of WR + the SILS port system was used for laparoscopic exploration of the abdomen.

The multiport technique was also used in this paper, and the total length of the incision was similar as well as the surgical time when only biopsies and aspirated were performed. The authors concluded that SILS laparoscopic-assisted GI procedures with a WR alone or combined with a SILS port was a safe technique for small intestinal exploration and targeted abdominal organ biopsy in cats.

Barry et al. [32] evaluated the feasibility of SILS versus open laparotomy for the diagnosis of specific lesions in dogs with suspected gastrointestinal obstruction. A SILS port system was placed through a 3-cm mini-laparotomy caudal to the umbilicus. After laparoscopic examination, the abdomen was decompressed, and the laparoscopic port was removed. The incision was enlarged by a Gelpi retractor or a wound retractor was placed in the surgical wound. The reported incision length was 4.9 cm for laparoscopy, with a surgical time of 36.8 min, and the respective incision length was 16.4 cm for the exploratory laparotomy, with a surgical time of 12.8 min. 

The authors concluded that, although the laparoscopy was feasible and clinically applicable in dogs with suspected gastrointestinal obstruction, careful patient selection and accurate evaluation of the conversion to an open surgical approach were recommended. According to the authors, dogs in critical conditions should not be considered suitable candidates for the SILS approach because it required significantly more time to perform than did exploratory laparotomy.

Otomo et al. [40] compared the outcomes in dogs that underwent SILS-assisted intestinal surgery and open laparotomy for simple foreign body removal using the SILS port. After the exploration, the single-incision port was removed, and the access incision was extended to a minilaparotomy of an approximate length of 5–7 cm centered on the umbilicus, which was retracted with WR. The reported surgical time of the surgery using SILS was 75 min for performing an enterotomy or enterectomy, while the median surgical time for open laparotomy was 60 min. The authors concluded that no significant differences were found in the surgical time and recovering in the post-operative time. 

The authors recommended that strict case selection be employed when deciding on the use of SILS for simple small intestinal foreign body removal in dogs and suggest the use of abdominal imaging technique as part of the preoperative diagnostic workup. Another consideration associated with the SILS port for GI surgical procedures was the location of the placement of the WR, reporting that a cranially placed WR may allow for an improved but not complete palpation of the stomach and remaining gastrointestinal tract providing excellent access to the jejunum, ileum, and cecum and may allow the ileocecocolic junction to be exteriorized.

Shamir et al. [41] described the surgical technique, biopsy sample quality, and short-term outcome of SILS small-intestinal exploration and abdominal organ biopsy using a WR device in 27 dogs. Laparoscopic exploration was performed through an SILS port in 18 patients in a surgery time of 86 ± 23 min; in five dogs, the multiple ports were employed completing the procedures in 116 ± 20 min; in four patients, a single 6-mm cannula was placed, resulting in a surgical time of 85 ± 9 min. The authors concluded that the use of WR provided an improved visualization of abdominal organs and reduced post-operative pain, compared with traditional retractors. 

Morris et al. [39] described a combination of WR and SILS ports for minimally invasive cisterna chyli ablation (CCA) evaluating this technique as a method for mesenteric lymphangiography in cases of idiopathic chylothorax. This hybrid technique performed in sternal recumbency allowed for both a CCA and an intraoperative lymphangiography through the same incision. 

### 3.4. Meta-Analysis

Of the 14 studies in which a control group was available, data extraction was possible in only four studies on ovariectomy (OVE) and four on gastrointestinal (GI) laparoscopic assisted surgery. Table 3 shows the specific articles and data extracted for meta-analysis.

**Figure 3:**
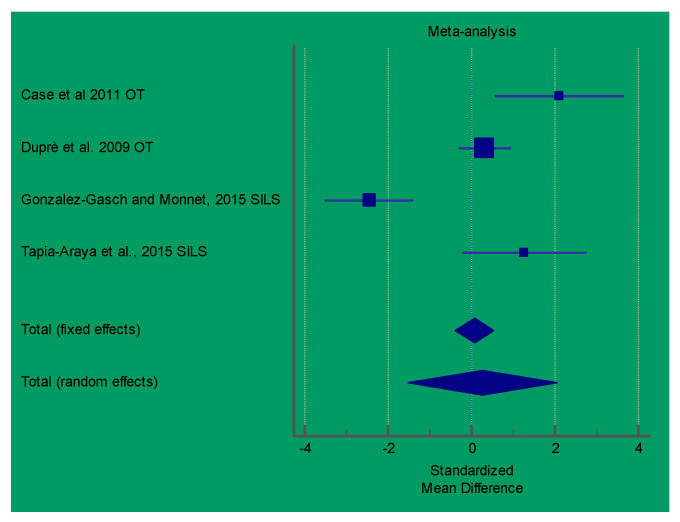
Forest-plot chart of the articles analyzed in terms of the surgical time of Ovariectomy (OVE) procedures. OT: operative telescope; SILS: SILS port platform. (Q 38.2976; I^2^ 92.17%; *p* < 0.0001).

**Figure 4:**
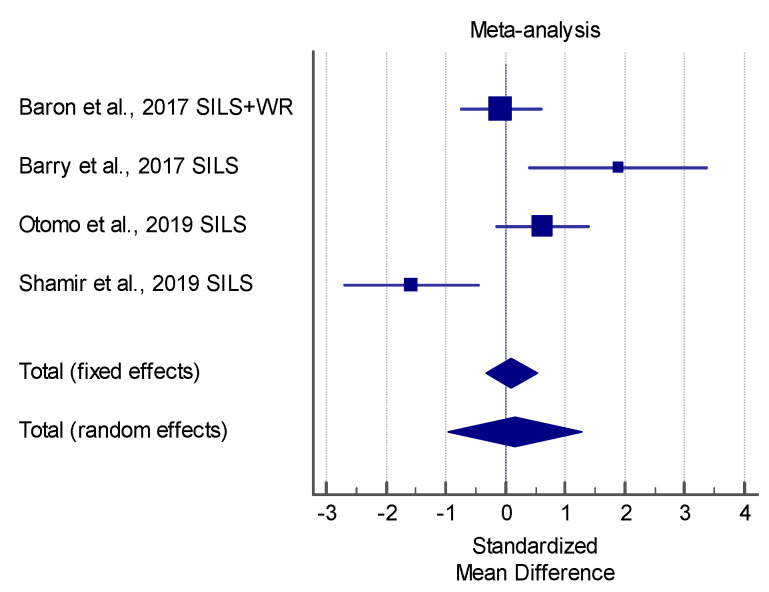
Forest-plot chart of the articles analyzed in terms of the surgical time of Gastrointestinal (GI) procedures. (Q 18.4577; I^2^ 83.75 %; *p* = 0.0004).

The results of the meta-analysis showed that the SILS techniques proposed in the study by Gonzlez-Gasch and Monnet [21] produced a significant lower surgery time in comparison with the control group (multiport), in performing OVE in dogs. In GI laparoscopic-assisted surgeries, the study by Shamir et al. showed a significantly lower surgical time relative to open surgery. However, the meta-analysis showed a very high heterogenicity (I^2^ > 90%) for both of the analyzed procedures. Figure 3 and Figure 4 show a forest plot graph of these articles. 

Unfortunately, data extraction for pain assessment and post-operative complications was not applicable, and a meta-analysis was not performed.

## 4. Discussion

The present review considered articles published from 2009 to 2020 on the use of SILS as minimally invasive procedures for different conditions in small animal surgeries. 

Considering the surgical times in the articles described in this report, most authors found that the surgical times using SILS were longer or not significantly shorter than those using multiport techniques in relation to reproductive tract surgery or laparoscopic prophylactic gastropexy. From the meta-analysis, the only report registering a significantly lower surgical time for SILS than for multiport techniques in OVE procedures was by Gonzalez-Gasch and Monnet [21] who described the use of a SILS port system. In human medicine, meta-analysis showed comparable operative times for colectomies, nephrectomies, and splenectomies [43,44,45] and increased operative times using a single-incision approach in appendectomies and cholecystectomies [46,47]. However, there is significant statistical heterogeneity in these comparisons, and all of these studies can be subject to self-selection bias [48].

The main cause of time consumption during SILS procedures was the loss of triangulation and instrument collisions. These notable technical difficulties also led to longer learning curves, which surgeons should consider when SILS procedures will be adopted, as opposed to the multiport techniques. The use of articulating instruments was described to reduce intraoperative difficulties [49]. However, flexible and steerable laparoscopic instruments need to be managed properly, and a learning curve should be expected by both novice and expert surgeons [50].

Studies on pain assessment are still very limited, and only some of the articles considered in this review properly or specifically examined pain in comparing SILS with multiport techniques. The study by Duprè et al. [3] described the use of OT and compared this SILS technique with the multiport system, finding that the pain in the two groups was comparable. Other studies in which pain assessment was evaluated were those of Case et al. in dogs [9] and Coisman et al. [17] in cats. In both studies, the pain level in the case of SILS was found to be similar to that in the case of multiport techniques. Binder et al. [34] reported a post-operative pain assessment, finding that SILS had the advantage of a low post-operative pain level, as evaluated by the owners as well as a very high owner satisfaction rate. Other data on pain were not available or were scarcely reported in the selected articles.

The main reported advantage of the use of the SILS port, compared to open celiotomy, was the ability of this technique to maintain a minimally invasive environment, allowing for a lower tissue trauma and fast post-operative recovery. Another advantage of the use of SILS was the possibility to suspend pneumoperitoneum and laparoscopic abdominal exploration, performing the rest of the surgical intervention extracorporeally. This ability was particularly appreciated during the GI tract surgery (i.e., enterectomy and enterotomy) [14,39,50] or uterus ligation during OVH [19,34,37] and Pyometra [15,24,26]. The combination of a WR and SILS port, or other systems that combine the removal of the cap with the instrument portal platform of WR, was particularly helpful for this purpose. 

In fact, removing the SILS port or the cap from the top of the WR, with the 360-degree hands-free wound retraction, allowed for the wound size to be minimized, with an improved exposure, and a distribution of force was even applied to the surgical wound, thus, maintaining moisture at the incision site and reducing surgical site infection. Specimen retrieval was also facilitated by the fact that the laparoscopic procedure could be easily interrupted, leaving the WR mounted on the surgical wound after completing organ resection of the ovary remnant, spleen, undescended testicles, tumors, or organ biopsied specimens.

The WR and SILS port require only a 2-to 4-cm incision length; however, correct positioning should be planned carefully to allow adequate visualization of the target organs or their exteriorization when needed [40]. A combination of WR with SILS or other WR-based platforms also prevents gas leakage, which is reported especially when using an Endocone or when an incision enlargement or two-portal connection requires organ exteriorization.

Since the SILS system placement requires unblinded access to the abdominal cavity through the mini celiotomy access, inadvertent abdominal organ puncture is avoided. SILS has significantly better outcomes in relation to cosmetic and body image in comparison to multiport techniques in human beings [51]. While, in small animals, this aspect should not be a primary concern, the owners’ overall satisfaction could be influenced by the surgical wound esthetic, especially in elective surgical procedures.

This study has certain limitations. In this review, only a few studies allowed data to be extracted for meta-analysis. Furthermore, these studies were conducted in limited populations. However, it must be considered that the SILS technique is an innovation and requires further studies to establish its real advantages. Furthermore, the studies included in this review were not uniform as they were conducted on different species, and the control groups were based on two- or three-port techniques; therefore, they are not free from bias.

Finally, although this study assessed the level of evidence of the included studies according to the previously described evaluation system, a critical judgment system, such as MINOR or other systems, was not used, adding further bias. However, a thorough evaluation was not the subject of our study, and the reader should bear in mind that further investigations should be conducted.

## 5. Conclusions

SILS techniques have been gaining ground in veterinary surgery over the past 10 years, following the wave of advances in minimally invasive surgery in the field of human surgery. However, the need to consider the aesthetic aspects associated with surgery for human surgery is far from being the same in the case of small animals, although it is still important.

The study of the articles available in the veterinary literature—although there is the presence of a large number of articles—does not allow for an adequate meta-analysis evaluation of the published results, especially regarding post-operative pain, evaluations of surgical times, and post-operative complications in comparison to multiport techniques. Therefore, veterinary surgeons who want to employ these techniques must consider that the real advantages of SILS techniques.

Especially in terms of pain levels, the maintenance of a minimally invasive environment and trauma to tissues can be guaranteed using multi-port techniques, which can reduce surgical times in a sensitive manner and do not involve the use of orientable tools or long learning curves. However, it should not be overlooked that SILS techniques have real advantages over open surgery and guarantee the execution of assisted laparoscopic procedures, while maintaining a minimally invasive environment, which contributes to a reduction of post-operative morbidity.

## Figures and Tables

**Table 1:** Levels of evidence criteria [7,8].

Study Design	Level of Evidence:
Randomized controlled trial	I
Prospective cohort study patients, compared with a control group of patients treated at the same time and institution	II
Case control study and retrospective cohort study	III
Case series (no control group or historical control group)	IV
Expert opinion	V

**Table 2:** Case series (CS); Randomized Clinical Trials (RCT); Retrospective Studies (RS); Clinical Trials (non-randomized, non-controlled) (CT); Controlled Clinical Trials (CCT); and Case Reports (CR). Level of Evidence: I, randomized controlled trial; II, Prospective cohort study patients, compared with a control group of patients treated at the same time and institution; III, Case control study and retrospective cohort study; and IV, case series (no control group or historical control group).

Author	Study Type	n Dogs	n Cats	Level of Evidence
Duprè et al., 2009 [3]	CCT	42		II
Case et al., 2011 [9]	RCT	18		I
Kim et al., 2011 [10]	CS		17	IV
Silva M.A. et al., 2011 [11]	RS	20		II
Manassero M. et al., 2012 [12]	CS	40		IV
Runge et al., 2012 [13]	CS	6		IV
Runge et al., 2013 [14]	RS	18		IV
Case J.B and Ellison G., 2013 [15]	CS	7	1	IV
Adamovich-Rippe et al., 2013 [16]	RS	12		IV
Coisman J.G et al., 2014 [17]	RCT		24	I
Naiman et al., 2014 [18]	RS	5	2	IV
Runge et al., 2014 [19]	RS	22	3	IV
Case et al., 2015 [20]	RCT		18	I
Gonzalez-Gasch and Monnet, 2015 [21]	RS	98		III
Rubin et al., 2015 [22]	CR	1		IV
Silva et al., 2015 [23]	CS	40		IV
Tapia-Araya et al., 2015 [24]	RCT	20		I
Wallace et al., 2015 [25]	CS	7		IV
Hoddinott et al., 2015 [26]	CR	1		IV
Becher-Deichsel et al., 2016 [27]	RS	10		IV
Gandini et al., 2016 [28]	CS	6		IV
Stiles et al., 2016 [29]	RS	14		IV
Wright et al., 2016 [30]	RS	18		IV
Baron et al., 2017 [31]	RS		42	III
Barry et al., 2017 [32]	CCT	16		II
Corriveau et al., 2017 [33]	RS	278		IV
Binder et al., 2018 [34]	RS	132		IV
Coutinho et al., 2018 [35]	RCT	24		I
Mayhew et al., 2018 [36]	RS	22		IV
Sakals et al., 2018 [37]	RCT		30	I
Bydzovsky et al., 2019 [38]	CT	42		IV
Morris et al., 2019 [39]	RS	14		IV
Otomo et al., 2019 [40]	RS	28		IV
Shamir et al., 2019 [41]	RS	27		III
Percival et al., 2020 [42]	RS	13		III
Total		1001	137	

**Table 3:** Data extracted for meta-analysis. (OVE: Ovariectomy; GI: Gastrointestinal).

Author and Instrument		Technique	Surgery Time SILS	SD SILS	N SILS		Surgery Time Control (Multiport)	SD Control	N Control
OVE									
Case et al. 2011	OT		29.7	5.6		6	18.2	4.4	6
Duprè et al. 2009	OT		21.07	6.25		20	19.06	6.24	22
Gonzalez-Gasch and Monnet, 2015	SILS Port		43	14		15	70	3	12
Tapia-Araya et al., 2015	SILS Port		36.6	3.5		5	32	3	5
GI Laparoscopic—assisted surgeries									
Baron et al., 2017	SILS Port + WR		82.5	0.6		22	84.2	21.1	15
Barry et al., 2017	SILS Port		36.8	0.96		13	12.8	1.2	3
Otomo et al., 2019	SILS Port		75	13.8		13	60	29.64	15
Shamir et al., 2019	SILS Port		86	23		18	116	20	5
